# Efficacy and safety of Soshiho-tang in patients with atopic dermatitis and gastrointestinal disorders: Study protocol for a double-blind, randomized, and placebo-controlled clinical trial

**DOI:** 10.1097/MD.0000000000015479

**Published:** 2019-05-03

**Authors:** Su-Jin Kang, Eun-Heui Jo, Geum-Jin Yang, Yu-Hwa Shim, Ji-Eun Hong, Min-Cheol Park

**Affiliations:** a Department of Korean Medicine Obstetrics & Gynecology, Won-Kwang University Korean Medicine Hospital, Iksan-si; b Department of Acupuncture and Moxibustion, Won-Kwang University Korean Medicine Hospital, Deokjin-gu, Jeonju-si; c Korean Medicine Dermatology Clinical Research Center of Won-Kwang University; d Department of Korean Medicine Ophthalmology and Otolaryngology and Dermatology, Won-Kwang University Korean Medicine Hospital, Iksan-si, Jeollabuk-do, Republic of Korea.

**Keywords:** atopic dermatitis, herbal medicine, randomized clinical trial, Soshiho-tang, study protocol, Syo-saiko-to, Xiao-chai-hu-tang

## Abstract

**Background:**

Atopic dermatitis (AD) is a chronically relapsing inflammatory skin disease that affects the quality of life in patients with AD. Since there is limitation of conventional treatment of AD, complementary treatment is required to treat AD symptoms more effectively and safely Soshiho-tang (SSHT) is a traditional herbal medicine that exhibits anti-inflammatory and anti-ulcer effects and improves the immune function. In this clinical trial, we will evaluate the efficacy and safety of SSHT in patients with AD and gastrointestinal disorders in comparison with placebo.

**Methods/design:**

This study is a single-center, randomized, double-blind, placebo-controlled, and investigator-initiated clinical trial. A total of 60 patients aged 3 to 18 years with AD and gastrointestinal disorders and who received a diagnosis of AD by Hanifin & Rajka criteria with a Scoring Atopic Dermatitis (SCORAD) index between 15 and 49 will be enrolled. Participants will be randomly assigned to the SSHT or placebo group in a ratio of 1:1. Additionally, they will have a visit schedule comprising 4 visits including a screening visit during 8 to 10 weeks. The participants will be administered SSHT or placebo 3 times a day for 4 weeks. The primary outcome will be measured by a change of the SCORAD index. The secondary outcome measures include the following: survey questionnaires for the perception of gastrointestinal disorders, amount and frequency of ointment usage for AD, dermatology quality of life index, itchiness and sleep disability score in visual analog scale, percutaneous water loss, skin surface temperature, Hamilton anxiety rating scale, and children's depression inventory.

**Discussion:**

In our knowledge, this will be the first clinical trial to assess the efficacy and safety of SSHT in patients with AD and gastrointestinal disorders. The findings of this study will provide new treatment options for patients with AD and gastrointestinal disorders.

**Trial registration:**

Korean National Clinical Trial Registry, Clinical Research Information Service. (KCT0003713) https://cris.nih.go.kr/cris/search/search_result_st01_en.jsp?seq=13489&ltype=&rtype=

## 1 Introduction

Atopic dermatitis (AD) is a chronically relapsing inflammatory skin disease which is characterized by pruritus, erythema, weeping from scratching, edema and lichenification.^[1,2]^ The worldwide prevalence of AD ranges from 5% to 20% among children aged 6 to 7 and 13 to 15 years and it is observed to be increasing in some parts of Africa, East Asia, and Western Europe for the last 20 years.^[3,4]^ With increasing prevalence of the disease, the socioeconomic burden of AD has been increasing.^[5]^

AD is a multifactorial disease caused by combination of mutations in the filaggrin gene, disrupted skin barrier, environmental factors, diet, and gut microbiota diversity.^[6,7,8]^ Although AD is not a life-threatening disease, children with AD often experience psychological and social difficulties including sleep disturbance, lack of concentration during the daytime, and isolation in school which can result in poor self-esteem, depression, frustration, as well as skin problems.^[9,10]^ It also has negative impact on the health-related quality of life of children with AD and their families.^[11,12]^ Moreover, 50% of children who were diagnosed with AD in their school-age show persistent AD in their adulthood.^[13]^

For the treatment of AD, topical corticosteroids, topical calcineurin inhibitors, barrier-enhancing creams, and systemic therapy are widely used depending on the severity of the symptoms.^[14]^ However, discontinuity in the treatment often results in relapse of the disease and several patients do not respond well to conventional treatments. Moreover, the use of long term systemic treatments can cause severe side effects including hyperglycemia, osteoporosis, renal toxicity, hypertension, and hepatotoxicity.^[7,15]^ Due to the limitations of conventional treatments, traditional herbal medicines have been increasingly used in the treatment of patients with AD.^[16]^

### 1.1 Soshihotang

Soshiho-tang (SSHT, Xiao-chai-hu-tang in China and Syo-saiko-to in Japan) is a traditional herbal medicine which is composed of 7 herbal components: Bupleuri Radix, Pinelliae Rhizoma, Rhizoma Zingiberis Recens, Scutellariae Radix, Zizhyphi Fructus, Glycyrrhizae Radix et Rhizoma, and Ginseng Radix.^[17]^ Although it has been primarily used to treat liver diseases including chronic hepatitis, hepatic cancer, and bronchial asthma,^[18]^ numerous clinical and experimental studies have demonstrated that SSHT exhibits anti-inflammatory effects by increasing the blood corticosterone level,^[19]^ improves immune function,^[20]^ and shows anti-ulcer effects by inhibiting gastric secretions.^[21]^ In our previous case report, we showed that SSHT displays the potential to treat AD with gastrointestinal disorders.^[22]^ Since there was no clinical trial, we designed a randomized, double-blinded, and placebo-controlled trial to investigate the efficacy and safety of SSHT on patients with AD and gastrointestinal disorders.

### 1.2 Study aims and hypothesis

The primary aim of this study is to evaluate the efficacy and safety of SSHT versus placebo for the treatment of AD patients with gastrointestinal disorders in randomized clinical trials. We hypothesize that SSHT is more effective than placebo for the treatment of AD patients with gastrointestinal symptoms. To assess the efficacy and safety of SSHT, a randomized, double-blinded, and placebo-controlled trial will be conducted.

## 2 Methods

### 2.1 Study design

**Figure 1:**
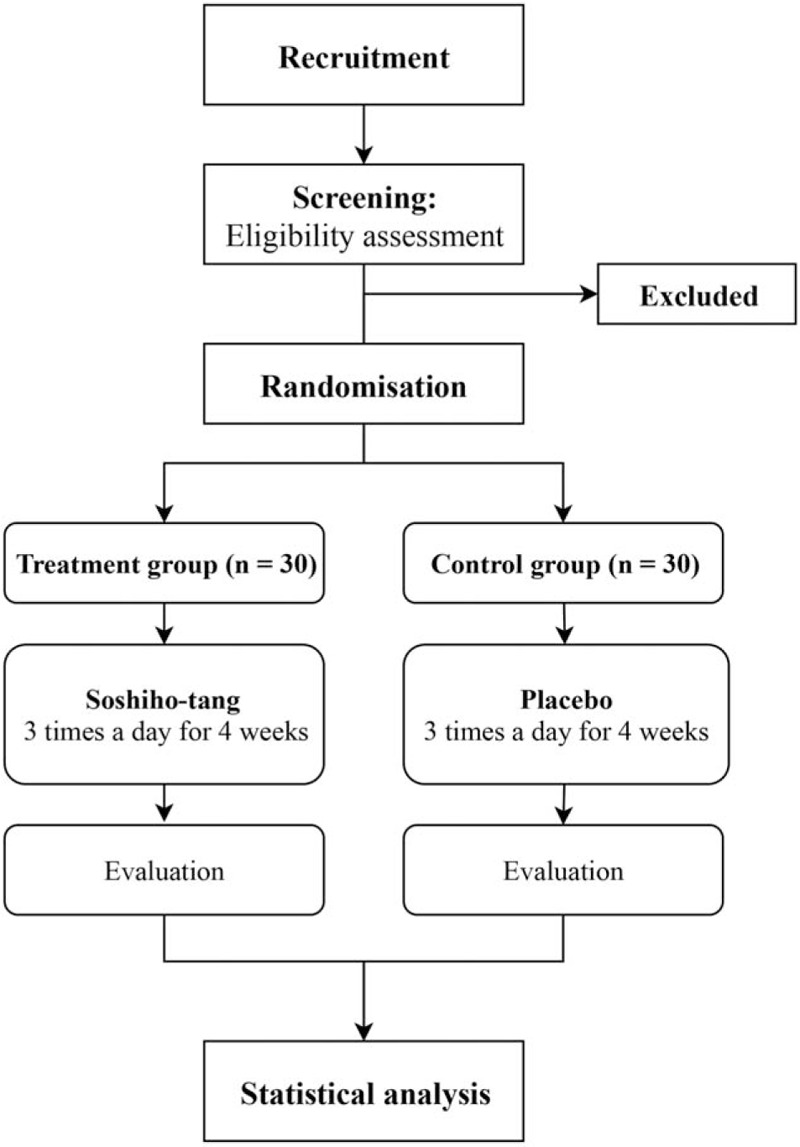
Study flowchart describing the details of the randomized controlled trial. The entire trial includes assessments on week 1, week 4, and week 8 (follow-up visit).

This clinical trial is designed as a single-center, randomized, double-blind, placebo-controlled, and investigator-initiated clinical trial. A total of 60 eligible participants will be divided equally into 2 groups: SSHT group and the placebo group. Participants will be administered SSHT or placebo 3 times day for 4 weeks. At weeks 4 and 8 (follow-up time points), participants will be instructed to visit the trial center for assessment of the efficacy and safety (including adverse events). The primary outcome for assessment of the efficacy and safety of SSHT is the Scoring Atopic Dermatitis (SCORAD) index, which is used to evaluate the severity of AD. The secondary outcome measures include the following: survey questionnaires for the perception of gastrointestinal symptoms, amount and frequency of ointment usage for AD, dermatology quality of life index, itchiness and sleep disability score in visual analog scale (VAS), percutaneous water loss, and skin surface temperature. The detailed schedule and assessments conducted throughout the clinical trial are summarized in Figure 1 and Table 1. The protocol (version 1.1, 8 April 2019) follows the Standard Protocol Items: Recommendations for Interventional Trials (SPIRIT) guidelines.

**Table 1:**
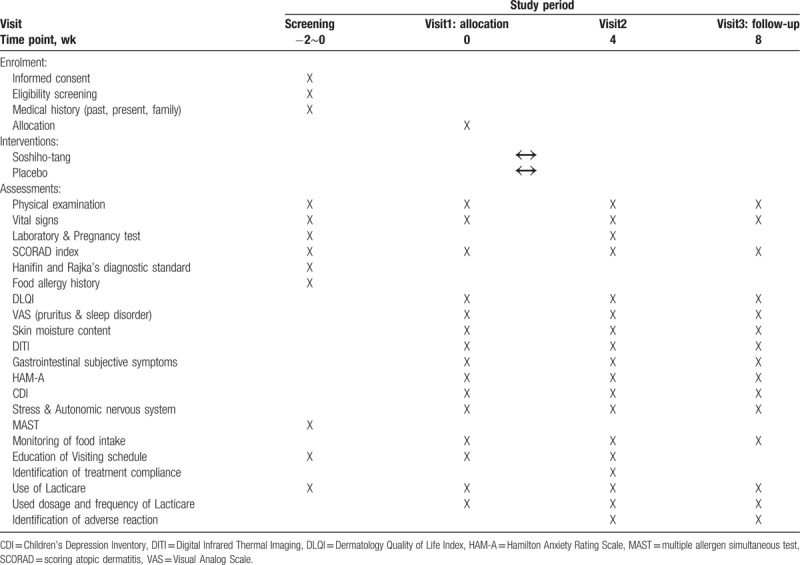
Schedule of enrolment, interventions, and assessments.

### 2.2 Participants

#### 2.2.1 Recruitment

Recruitment of the participants began in September 2017 at the Won-Kwang University Korean Medicine Hospital and is expected to finish in August 2020. The advertisement to recruit participants for this clinical trial includes notices on bulletin boards in commercial buildings, apartments, hospitals, and on the streets. Written consent will be obtained from all participants before registration, and participants may withdraw from the trial at any point in time without penalties. The written consent form includes information regarding the background and purpose of the study, trial drug and placebo drug, outcome, and the expected benefits and drawbacks.

#### 2.2.2 Inclusion criteria

Participants should meet all of the following inclusion criteria:

1. Males and females aged 3 to 18 years with SCORAD scores between 15 and 49, exhibiting ≥3 main and ≥3 accessory symptoms based on the diagnostic criteria developed by Hanifin and Rajka
2. Participants with ≥1 box checked for gastrointestinal symptoms or food allergy history, in the questionnaire survey for gastrointestinal disorders
3. Participants who have fully understood the information provided about the study voluntarily decided to participate and agreed to comply with the precautions (written consent is required from the participants’ parents)

#### 2.2.3 Exclusion criteria

1. Patients receiving treatment for severe AD (antihistamines, adrenocortical hormones, Korean traditional herbal medication)
2. Patients who have previously used oral antihistamines, steroids, antibiotics, systemic photochemotherapy, and other immune suppressants in the previous 4 weeks
3. Patients with systemic infections or under treatment with systemic antibiotics
4. Patients with severe dermatological disorders aside from AD, pigmentation, or large scarring of the area affected by AD
5. Patients currently receiving interferon (IFN) drug treatments (IFN-α, IFN-β)
6. Patients with liver diseases (liver cirrhosis, liver cancer)
7. Patients with platelet count ≤100,000/mm^3^ due to chronic hepatitis-induced liver dysfunction
8. Patients with kidney diseases (acute/chronic renal failure, renal syndrome)
9. Patients with acute severe cardiovascular diseases (cardiac failure, myocardial infarction, cerebral infarction)
10. Patients with a history of antipsychotic drug use within 2 months before the screening examination
11. Patients who have participated in other clinical trials within the past 4 weeks
12. Patients with a history of hypersensitivity or allergic reaction to an ingredient of the clinical trial product or the product itself
13. Patients with a history of drug or alcohol abuse
14. Patients who are pregnant or breastfeeding
15. Patients who may become pregnant and have not used appropriate contraceptives (excluding those who have undergone sterilization surgery)
16. Patients who are judged ineligible to participate in the trial by the principal investigator for other reasons, including diagnostic radiology outcomes

### 2.3 Study phase

When patients visit the trial center for the screening process, they will undergo different assessments, including blood and urine tests, and answer a survey questionnaire on AD symptoms by which the investigator may determine the patient's eligibility to participate in this clinical trial. At the screening visit, Lacticare (hydrocortisone-acetate 1% (100 g)) will be provided as a control variable which is the conventional therapy of AD to all participants. The amount and frequency of ointment usage from the screening visit to visit 3 (8-week time point) will be recorded. Patients found eligible to participate will be informed of their eligibility via a telephone call, and selected participants will be asked to visit the trial center (visit 1) within 2 weeks of the screening examination for random assignment into either the experimental group or the control group. After the assignment, the participant will undergo additional assessments (answering survey questionnaires including those on AD, as well as a physical examination) and will receive enough of the trial product for 4 weeks (to be used 3 times per day). After 4 weeks, participants will be asked to return for visit 2 to perform identical assessments (survey questionnaires and blood tests). For the following 4-week period, the participants will be asked to stop the usage of all medication, with the exception of Lacticare, and return for visit 3 after 4 weeks to undergo the same assessment. Detailed assessment types and the schedule used are outlined in Table 1.

### 2.4 Intervention

Participants in the experimental and control groups will be required to take SSHT or the placebo product, respectively, for 4 weeks, 3 times a day before each meal. The dosage for different age groups is as follows: 4.5, 6, and 9 g/day for participants aged 3 to 5, 6 to 13, and 14 to 18 years, respectively. Both the SSHT and the placebo were manufactured by Hanpoong Pharm. Co Ltd., according to Korea Good Manufacturing Practice standards. Notably, both SSHT and placebo are of identical color, dosage form, and appearance (dark brown granules). The SSHT consists of 2.33 g Bupleuri Radix, 1.7 g Pinelliae Rhizoma, 1.33 g Zingiberis Rhizoma Recens, 1.00 g Scutellariae Radix, 1.00 g Zizhyphi Fructus, 1.00 g Ginseng Radix, 0.67 g Glycyrrhizae Radix et Rhizoma, 1.31 g lactose hydrate, and 0.315 g corn starch. The placebo consists of 2.57 g corn starch, 0.358 g lactose hydrate, 0.05 g citric acid hydrate, 0.02 g caramel colorant, and 0.0002 g ginseng flavor powder. Both SSHT and the placebo groups will receive Lacticare (hydrocortisone-acetate 1% (100 g)) for the entire duration of the trial from the screening point (12 weeks) as a control variable.

### 2.5 Randomization

Participants who provide written consent to participate in this clinical trial will be assigned screening numbers in the chronological order of consent received. Participants deemed eligible according to the inclusion/exclusion criteria will be assigned a random number in the order of their visit date. Additionally, the Strategic applications software (SAS, software version 9.3) will be used to assign the participants randomly to either the experimental group or the control group. A method of block randomization, involving the use of a set assignment ratio (1:1) within identically sized blocks (i.e., 4, 6, or 8 participants per block), will be used. Before the start of the clinical trial, the research pharmacist at the trial center will provide the participants with a box containing the trial product, and the number on the box will match the participant's random assignment number.

### 2.6 Blinding

In order to obtain reliable data from the clinical trial, double-blinding is performed so that neither the investigator nor the participants know which participant is assigned to which group. Double-blinding will be maintained unless a medical emergency occurs. Data locking will be maintained for information regarding the group assignment and will be made publicly available at the time of statistical analysis after the completion of the clinical trial. In case of cessation of double-blinding for different reasons, such as an adverse event, the appropriate procedure will be followed to report the incident to the Institutional Review Board (IRB) and to notify the sponsor.

### 2.7 Sample size calculation

As the primary outcome to evaluate the efficacy of SSHT in patients with AD experiencing gastrointestinal disorders, this study will use the SCORAD scores. We calculated the sample size based on the previous study which evaluated the efficacy of Shiho Cheong gan-san for patients with AD.^[23]^ It is hypothesized that the mean changes of the SCORAD score in the SSHT and placebo groups after 4 weeks of intervention were 13.8 (μt) and 7.9 (μ*c)*, respectively and the standard deviations of the SCORAD scores between 2 groups were 8.2. A total of 60 participants, 30 in each group, are needed to achieve a statistical power of 80%, significance level of 0.05 in 2-tailed test, based on an estimated 20% dropout rate.

### 2.8 Statistical analysis

Appropriate statistical analysis methods will be used according to the type of measurement, number of associated factors and associations, as well as the distribution status. The software that will be used for statistical analysis is the SAS version 9.3 for Windows, at a statistical significance level of 0.05 and statistical power of 80%.

### 2.9 Efficacy assessment

As assigned, the Intent-to-treat (ITT) group comprises participants who will consume the trial product at least once, from the time of random assignment. The data for efficacy assessment will be mainly based on the full analysis set (FAS) group. However, additional analysis will be performed on the per protocol (PP) group. The FAS group is composed of participants who will consume the trial product after randomization and undergo at least 1 recorded measurement of primary efficacy assessment variables. On the other hand, the PP group is composed of all ITT participants who will complete the clinical trial without major violations of the study protocol.

For efficacy assessment, paired *t-*tests will be performed to compare measurements at weeks 4 and 8 to those recorded at baseline in order to assess the statistical significance of the changes within the treatment group. Inter-group comparisons will be performed via an analysis of covariance with an adjusted baseline. In order to assess the differences between the 2 groups with regard to trend changes after each visit, a repeated measures analysis of variance will be performed. Differences within groups before and after the intervention will be comparatively assessed via either paired *t*-tests or Wilcoxon signed rank tests.

### 2.10 Safety assessment

The data for safety assessment of the trial product will be based on the safety group. The safety population (SP) group is composed of all participants who will undergo at least 1 intervention and post-intervention safety assessment.

- Adverse events: either the Chi-square or Fisher exact test will be performed to compare the differences between groups, and a summary sheet for each body organ will be created. In addition, the incidence rate of adverse events that are confirmed to be associated with either the trial product or the placebo product will be comparatively assessed using the same method.
- Laboratory test values: after completion of the trial, the fraction of incidences that exhibit clinical test values exceeding the normal range will be assessed using either Chi-square or Fisher exact test.
- Vital signs: means and standard deviations for blood pressure and heart rate will be recorded at each visit. Furthermore, significant changes in the categories of vital signs between the start and the end of the intervention will be assessed using either paired *t* tests or Wilcoxon signed rank tests.

### 2.11 Data management

Information obtained from the visit and examination of each participant will be recorded on a paper print-out. The information will then be hand-written on a paper document case report form (CRF) and entered into an Excel file for future statistical analyses. All of the above information will be stored and managed by the Center for Atopic Dermatitis Research, Iksan Korean Medicine Hospital, Won-Kwang University. In compliance with the Personal Information Protection Act, the names of all participants will not be disclosed and a unique identifier number given during the trial will be used to identify participants. All the participants will be informed that the clinical data obtained in the trial will be stored in a computer and will be handled with confidentiality. The participants’ written consent will be stored by the principal investigator.

### 2.12 Monitoring

A Contract Research Organization (CRO) specialized in monitoring will perform the overall monitoring of the clinical trial, in order to ensure the quality, accuracy, and reliability of the clinical trial and data obtained. The monitoring agent will compare the source data written on the CRF with those on the source document for source data verification. Protocol required data, which are required to fulfill the requirement of the International Conference on Harmonization's Good Clinical Practices (ICH-GCP), will be reported accurately and consistent with the source. In addition, the following aspects will be monitored: modifications of dose or therapy, adverse events, concomitant medications history, intercurrent illnesses, withdrawals, and dropouts. Monitoring visit reports will include a summary of the visit, descriptions, issues, and checklist results.

### 2.13 Safety and adverse events

In this clinical trial, the participants will undergo blood and urine tests during the initial screening visit (before consumption of the trial product) and at week 4 (4 weeks after initial consumption of the trial product) for safety assessment. In order to investigate the adverse events, the participants will be asked to return for an in-person interview at week 8 (1 month after the end of the trial product consumption) as a follow-up assessment of adverse events. In the case of adverse events, the principal investigator will record all relevant information (including the dates for the start and end of the adverse event, the severity, association with the trial product and associated interventions, treatment, and outcome) on the CRF. In addition, all clinically significant abnormal findings in laboratory tests or vital sign assessments will be recorded as adverse events.

### 2.14 Ethics

This clinical trial will be performed in compliance with the Declaration of Helsinki, ICH-GCP guidelines, and all other applicable domestic and international regulations. In addition, IRB approval was obtained from the clinical research ethics committee of the Iksan Korean Medicine Hospital, Won-Kwang University (OMH-IRB-11V1.1). Any protocol modifications will be approved by the IRB of the Won-Kwang University Korean Medicine Hospital. SSHT was approved for the treatment of AD by the Korean Ministry of Food and Drug Safety.

## 3 Discussion

This is the study protocol to assess the efficacy and safety of SSHT on AD patients with gastrointestinal disorders in a randomized, double-blinded, and placebo-controlled trial. We will evaluate the efficacy and safety of SSHT compared to placebo after 4 weeks of treatment and we will recruit patients aged from 3 to 18 years and who have AD and gastrointestinal disorders. Although it has been acknowledged that AD starts during the first year of life and remits before school age, recent study reported that 50% of children with early onset of AD suffered relapsing AD from childhood to adulthood.^[13]^ Consequently, we considered it important to focus on AD patients before their adulthood.

As a control variable, Lacticare (hydrocortisone-acetate 1% (100 g)) will be given to both the SSHT and placebo groups from the screening point until the end of intervention. We have set the control variable to apply minimum treatment for the placebo group patients. For an accurate analysis of the efficacy of SSHT, the amount and count of used ointments will be recorded strictly.

Although AD is a complex multifactorial disease, it is acceptable that food hypersensitivity has a pathogenic role in the etiology of AD.^[24,25]^ The prevalence of food allergies among AD patients is higher compared to that of the general population.^[26]^ Food allergens are shown by immunoglobulin E mediated immediate-type reactions including gastrointestinal, respiratory, and urticarial symptoms and it is easy to cause eczema in children with AD.^[27]^ In this study, we will examine the multiple allergen simultaneous test (MAST), Ig-E, and questionnaire of gastrointestinal disorders to decide whether patients have food allergies. As it is known that SSHT displays anti-inflammatory and immunomodulating activities,^[28]^ we anticipate that this traditional herbal medicine will be effective on both AD and gastrointestinal disorders.

This study is the first randomized clinical trial to evaluate the efficacy and safety of SSHT versus placebo for the treatment of patients with AD and gastrointestinal disorders. The outcome of this study will provide a new strategy for the treatment of AD with gastrointestinal disorders.

## Author contributions

**Conceptualization:** Su-Jin Kang, Min-Cheol Park.

**Data curation:** Yu-Hwa Shim.

**Methodology:** Eun-Heui Jo.

**Project administration:** Min-Cheol Park.

**Resources:** Su-Jin Kang, Yu-Hwa Shim, Ji-Eun Hong.

**Supervision:** Min-Cheol Park.

**Visualization:** Geum-Jin Yang.

**Writing – original draft:** Su-Jin Kang.

**Writing – review & editing:** Min-Cheol Park, Eun-Heui Jo, Geum-Jin Yang, Ji-Eun Hong.
